# Arch enemy no more: designing the first synthetic globular all-beta proteins with beta-arches

**DOI:** 10.1093/synbio/ysz002

**Published:** 2019-02-04

**Authors:** Daniel Bojar

**Affiliations:** 4058 Basel, Switzerland

*De novo* protein design, taking its first steps at the turn of the century (1), aims to create novel proteins without an existing protein scaffold wholly from foundational structural principles and simulations. These designed proteins often have sequences and functions that are unlike anything found in nature and can even exhibit completely novel protein folds. The wholesale design of new proteins thus requires an exquisite understanding of their 3D structure. Thanks to their modular properties, proteins consisting solely of alpha-helices were the first to yield to protein design *de novo*. In a recent publication in the journal *Nature Structural & Molecular Biology*, David Baker’s team at the University of Washington made a foray into uncharted territory and for the first time succeeded in designing a globular all-beta protein *de novo* (2).

To understand their success, it is paramount to understand why it is so difficult to design globular proteins consisting only of beta-sheets. In contrast to amino acid residues in alpha-helices, which establish most of their contacts with residues nearby, residues in beta-sheets frequently interact with residues that are farther apart on the sequence, making them harder to design. For example, beta-arches are loops that connect two beta-strands that do not form a continuous beta-sheet. In this publication, the authors discovered and utilized structural principles of all-beta proteins to facilitate their design process. These principles for instance constrained the geometry of beta-arches by assigning amino acid frequencies as well as the orientation of their side chains to specific types of beta-arches.

Led by researchers Enrique Marcos and Tamuka M. Chidyausiku, the team used the protein modeling software Rosetta to construct candidates for globular all-beta proteins with differing lengths of beta-strands and beta-arches. Choosing 19 of these final jellyroll protein structure candidates consisting of eight antiparallel beta-strands for experimental characterization, they recombinantly expressed them in *E. coli*. Obtaining an nuclear magnetic resonance (NMR) spectroscopy structure of one of these expressed designer proteins, the authors could demonstrate that the actual protein structure closely resembled their *de novo* design. While proteins consisting of beta-strands connected by tight loops (effectively forming a flowing carpet of paired beta-strands) have been attempted before, the *de novo* design of beta-arches is definitely a novelty. This characteristic allows all-beta proteins to fold into globular proteins, in contrast to the previous elongated designs, and is needed for complex proteins such as antibodies.

Why is all of this important? In addition to a profound conceptual leap in our ability to design proteins *de novo*, this work furthers our structural understanding of all-beta proteins. Many important proteins, such as the nucleosome-chaperone nucleoplasmin and many viral capsid proteins, contain jellyroll folds. This publication brings us closer toward the aspirational goal of understanding natural proteins and building novel proteins that contain these complex 3D structures.
